# Prognostic effects of cardiopulmonary resuscitation (CPR) start time and the interval between CPR to extracorporeal cardiopulmonary resuscitation (ECPR) on patient outcomes under extracorporeal membrane oxygenation (ECMO): a single-center, retrospective observational study

**DOI:** 10.1186/s12873-023-00905-8

**Published:** 2024-03-05

**Authors:** Amir Vahedian-Azimi, Ibrahim Fawzy Hassan, Farshid Rahimi-Bashar, Hussam Elmelliti, Mahmood Salesi, Hazim Alqahwachi, Fatima Albazoon, Anzila Akbar, Ahmed Labib Shehata, Abdulsalam Saif Ibrahim, Ali Ait Hssain

**Affiliations:** 1 Trauma research center, Nursing Faculty, Baqiyatallah University of Medical Sciences, Tehran, Iran; 2 Medical Intensive Care Unit, Hamad General Hospital, Doha, Qatar; 3 Department of Medicine, Weill Cornell Medical College, PO BOX 3050, Doha, Qatar; 4 Department of Anesthesiology and Critical Care, School of medicine, Hamadan University of Medical Sciences, Hamadan, Iran; 5 Emergency Department, Hamad General Hospital, Doha, Qatar; 6 Chemical Injuries Research Center, Systems Biology and Poisonings Institute, Baqiyatallah University of Medical Sciences, Tehran, Iran; 7 Medical Education, Hamad Medical Corporation, Doha, Qatar; 8 Medical Research Center, Hamad Medical Corporation, Doha, Qatar

**Keywords:** Cardiac arrest, Cardiopulmonary resuscitation, Extracorporeal circulation, Extracorporeal membrane oxygenation out-of-hospital cardiac arrest, In-hospital cardiac arrest, Prognosis

## Abstract

**Background:**

The impact of the chronological sequence of events, including cardiac arrest (CA), initial cardiopulmonary resuscitation (CPR), return of spontaneous circulation (ROSC), and extracorporeal cardiopulmonary resuscitation (ECPR) implementation, on clinical outcomes in patients with both out-of-hospital cardiac arrest (OHCA) and in-hospital cardiac arrest (IHCA), is still not clear. The aim of this study was to investigate the prognostic effects of the time interval from collapse to start of CPR (no-flow time, NFT) and the time interval from start of CPR to implementation of ECPR (low-flow time, LFT) on patient outcomes under Extracorporeal Membrane Oxygenation (ECMO).

**Methods:**

This single-center, retrospective observational study was conducted on 48 patients with OHCA or IHCA who underwent ECMO at Hamad General Hospital (HGH), the tertiary governmental hospital of Qatar, between February 2016 and March 2020. We investigated the impact of prognostic factors such as NFT and LFT on various clinical outcomes following cardiac arrest, including 24-hour survival, 28-day survival, CPR duration, ECMO length of stay (LOS), ICU LOS, hospital LOS, disability (assessed using the modified Rankin Scale, mRS), and neurological status (evaluated based on the Cerebral Performance Category, CPC) at 28 days after the CA.

**Results:**

The results of the adjusted logistic regression analysis showed that a longer NFT was associated with unfavorable clinical outcomes. These outcomes included longer CPR duration (OR: 1.779, 95%CI: 1.218–2.605, *P* = 0.034) and decreased survival rates for ECMO at 24 h (OR: 0.561, 95%CI: 0.183–0.903, *P* = 0.009) and 28 days (OR: 0.498, 95%CI: 0.106–0.802, *P* = 0.011). Additionally, a longer LFT was found to be associated only with a higher probability of prolonged CPR (OR: 1.818, 95%CI: 1.332–3.312, *P* = 0.006). However, there was no statistically significant connection between either the NFT or the LFT and the improvement of disability or neurologically favorable survival after 28 days of cardiac arrest.

**Conclusions:**

Based on our findings, it has been determined that the NFT is a more effective predictor than the LFT in assessing clinical outcomes for patients with OHCA or IHCA who underwent ECMO. This understanding of their distinct predictive abilities enables medical professionals to identify high-risk patients more accurately and customize their interventions accordingly.

## Background

Cardiac arrest (CA) is a life-threatening condition characterized by the sudden cessation of effective heart function, leading to an interruption in blood flow and oxygen supply to vital organs [1]. Despite advances in medical science and resuscitation techniques, CA remains a significant global public health concern with high mortality rates [2]. The survival rate with favorable neurological outcome for out-of-hospital cardiac arrest (OHCA) ranges from 5 to 10% [3, 4]. In-hospital cardiac arrest (IHCA) has a slightly better prognosis, with survival rates ranging from 15 to 25% [1, 5]. However, it is crucial to emphasize the importance of treating these patients as soon as possible. Delaying the initiation of resuscitation decreases the chances of survival and increases the risk of neurological damage [6, 7].

Prompt initiation of cardiopulmonary resuscitation (CPR) is vital for maintaining tissue perfusion and increasing the chances of successful resuscitation [8, 9]. Every minute of delay in providing CPR and defibrillation correlates with a 7–10% decrease in survival [10, 11]. However, in some cases, conventional CPR (C-CPR) may not be sufficient in achieving a sustained return of spontaneous circulation (ROSC), and extracorporeal cardiopulmonary resuscitation (ECPR) may be necessary [12, 13]. ECPR involves the use of extracorporeal membrane oxygenation (ECMO) to provide circulatory and respiratory support, and it has been shown to improve survival rates and neurological outcomes in patients with refractory CA [14, 15]. However, the optimal timing and duration of CPR before transitioning to extracorporeal cardiopulmonary resuscitation (ECPR) are still areas of active research and debate.

Evidence indicates that the duration of total collapse, which refers to the time from CA to the ROSC as well as to the ECPR implementation in patients experiencing refractory CA, plays a crucial role in predicting patient outcomes for both OHCA and IHCA [16–20]. The total collapse duration encompasses two components: the time from collapse to the initiation of CPR, known as the “no-flow time” (NFT), and the time from CPR to ROSC referred to as the “low-flow time” (LFT) [21]. A meta-analysis has demonstrated that a shorter LFT is independently associated with more favorable neurological outcomes in individuals who undergo ECPR [22]. Furthermore, a study suggested that NFT and LFT interact and influence clinical outcomes in OHCA patients [23]. However, the precise impact of NFT and LFT on clinical outcomes, particularly neurological outcomes, remains unclear for CA patients who receive ECPR. Multiple studies have examined the prognostic significance of NFT and LFT in ECPR recipients, but their findings have been inconsistent. While some studies have found that shorter NFT and LFT are linked to better outcomes, others have reported no significant association [24, 25].

Thus, to address this issue, we conducted this study to investigate the prognostic effects of NFT and LFT on ECMO patient outcomes. Our hypothesized that shorter NFT and LFT intervals would be associated with better outcomes in patients who received ECPR. Our study adds to the existing literature on the prognostic impact of NFT and LFT on ECPR outcomes and may help inform clinical decision-making in the management of patients with refractory CA.

## Methods

### Study design and ethical approval

This single-center, retrospective observational study was conducted at Hamad General Hospital (HGH), the tertiary governmental hospital of Qatar, between February 2016 and March 2020. The aim of the study was to evaluate the impact of two-time intervals on the outcomes of patients with both OHCA and IHCA who underwent ECPR. The study adhered to the principles of the Declaration of Helsinki [26], and received approval from the Clinical Investigation Ethics Committee of Hamad General Hospital Institutional Review Board (MRC-01-21-934). Informed consent was obtained from all patients or their legal guardians. The study findings were reported in accordance with the Strengthening the Reporting of Observational Studies in Epidemiology (STROBE) guidelines [27].

### Study population

**Fig. 1:**
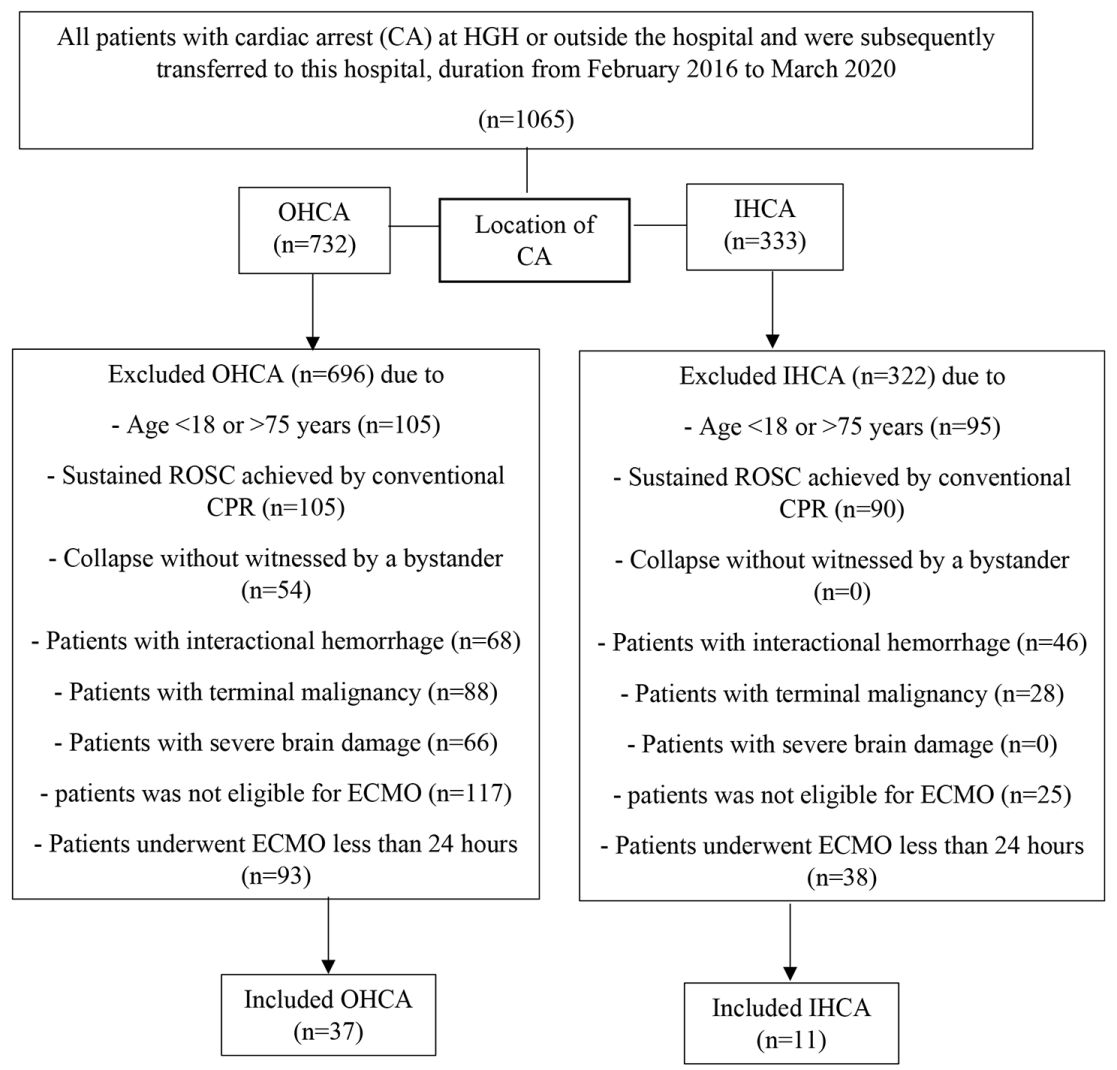
Flow diagram of the study population. Abbreviations: OHCA; out-of-hospital cardiac arrest, IHCA, in-hospital cardiac arrest, ECMO; extracorporeal membrane oxygenation

All adult patients (≥ 18 years) who experienced a cardiac arrest at HGH or outside the hospital and were subsequently transferred to this hospital were eligible to enroll in this study if they met all inclusion criteria. The enrollment criteria for participants in this study included: (a) Cardiac arrest patients aged 18 years or older, regardless of whether the cardiac arrest occurred in-hospital or out-of-hospital; (b) Cardiac arrest patients in whom return of spontaneous circulation (ROSC) could not be achieved by conventional CPR (sustained ROSC was defined as the absence of additional chest compressions for at least 20 min); (c) Patients who received ECMO for refractory out-of-hospital cardiac arrest (OHCA) and in-hospital cardiac arrest (IHCA), including veno-venous ECMO (VV-ECMO) or veno-arterial ECMO (VA-ECMO), for at least24 hours; (d) Collapse witnessed by a bystander or reliable report of estimated collapse time. However, patients who met at least one of the exclusion criteria were not included in the present analysis. These exclusion criteria included: (a) patients aged 75 years or older; (b) patients with ongoing intracranial hemorrhage; (c) patients with terminal malignancy; (d) patients who required constant support; (e) patients with cardiac tamponade caused by aortic dissection; and (f) those with evidence of severe brain damage. The flow diagram depicting the study population is presented in Fig. 1.

### Procedure for ECMO

A specialized team of medical professionals, including a cardiac anesthesiologist, a cardiac surgeon, a perfusionist, and a nurse from the cardiac surgical ICU, provided treatment to all patients experiencing IHCA or OHCA. The team followed the American Heart Association (AHA) 2016–2020 CPR guidelines [28]. Patients were selected for ECMO based on the assessment of the resuscitation medical team, taking into consideration various criteria such as: (a) presence of witnessed arrest, (b) initial findings from the electrocardiogram (ECG) (the initial findings from the ECG in ECMO are important for diagnosis, helping identify pre-existing cardiac conditions and guiding the decision to initiate ECMO. Additionally, the initial ECG serves as a baseline assessment, allowing clinicians to monitor changes in the heart’s electrical activity and detect any new abnormalities throughout the ECMO course), (c) identification of a reversible cause of cardiac arrest, (d) hospital arrival within ≤ 45 min for out-of-hospital cardiac arrest (OHCA) patients, and (e) administration of bystander CPR [29, 30]. The process of ECMO initiation for the patients followed a specific protocol. Cannulation was promptly performed at the patient’s current location. In the case of patients with OHCA, the ECMO team would wait for their arrival at the emergency room, where the necessary equipment for vessel cannulation had been prepared in advance. However, for patients with IHCA, the resuscitation team was present at their bedside. During cannulations, either the cardiac anesthesiologist or the cardiac surgeon performed the procedure, while the perfusionist assembled the circuit and primed it with Ringer’s lactate solution. After placing the arterial cannula, a heparin bolus of 50 units per kilogram is promptly administered to the patient. Continuous intravenous infusion of heparin is then maintained to achieve and sustain an activated clotting time of 160–180 s. Once ECMO was initiated, the patient would be transferred to the catheter laboratory if cardiac causes were suspected, or to the cardiac surgery operating room. Adjustments to the ECMO blood flow would be made to maintain a cardiac index of 2.6 L per minute per square meter or higher, with the goal of achieving an inlet venous saturation above 70%. To provide brain protection following cardiac arrest, all patients received hypothermic treatment (32–34 °C) within the first 24 h whenever possible. The Cardiohelp ©system (Maquet, Rastatt, Germany) was utilized for the treatment of all patients. The preferred technique involved percutaneous cannulation using the Seldinger technique through the femoral vein and femoral artery (cannula sizes: Access Cannula: 21–23 FR, Return Cannula: 15–17 FR, Reperfusion Cannula: 7–8 FR). In cases where peripheral cannulation was not feasible, central cannulation took place in the cardiac surgery operating room, involving access through the right atrium and ascending aorta (cannula sizes: DLP 20–22 Fr and 51 Fr, Medtronic Inc., Minneapolis, MN, USA). The dimensions of the catheters were selected based on the patient’s body size. To further enhance the system, the tubing, pump, and oxygenator were coated with Bioline® coating.

### Data collection

Data on IHCA and OHCA were collected by trained observers during both day and night shifts. For IHCA, the observers accompanied the cardiac arrest team but were not directly involved in the treatment. Prehospital data for OHCA was collected by emergency medical services (EMS). In addition, observers working with the cardiac arrest team gathered data on in-hospital resuscitation procedures and treatments.

Demographic and clinical information for patients was obtained from their medical records. This information included details such as age, gender, nationality, body mass index (BMI), presence of comorbidities, severity of illness (based on Acute Physiology and Chronic Health Evaluation [APACHE-II] [31] and Sequential Organ Failure Assessment [SOFA] [32] scores), location of cardiac arrest (out-of-hospital or in-hospital), and the cause of cardiac arrest (e.g., ischemic heart disease, pulmonary embolism, or other factors).

For OHCA, data on prehospital characteristics included the time of collapse, presence of witnesses, bystander CPR, initial shockable rhythm, time intervals between collapse and the start of CPR, duration of CPR, and time intervals between CPR and the initiation of ECPR. As for IHCA patients, the attending physician utilized a standardized data collection form to document the resuscitation process. Investigators who were not directly involved in the patients’ care gathered outcomes related to ECMO (such as ECMO type, length of stay, and survival), along with the length of stay in the intensive care unit (ICU) and hospital, disability status at 28 days (based on the Modified Rankin Scale), neurological status at 28 days (based on the Cerebral Performance Category), as well as survival at 24 h and 28 days.

### Definition and research instruments

A shockable rhythm refers to ventricular fibrillation or pulseless ventricular tachycardia [33, 34]. The no-flow time (NFT) represents the interval from collapse to the initiation of the resuscitation process, typically through CPR [21, 35]. additionally, the low-flow time (LFT) represents the duration from the start of CPR to the implementation of extracorporeal CPR (ECPR) [21, 36].

To assess disability and neurological status 28 days after cardiac arrest (CA), two scales were employed. The Modified Rankin Scale (mRS) [37], which consists of seven grades ranging from 0 to 6, was used to evaluate overall outcome and functional status. Grade 0 signifies no symptoms, grade 1 indicates no significant disability but potentially minor symptoms, grade 2 reflects slight disability with limitations in certain activities, grade 3 represents moderate disability with assistance required for some activities but independent walking capability, grade 4 implies moderately severe disability requiring supervision and assistance for most activities, grade 5 denotes severe disability necessitating constant assistance and care due to substantial functional impairment, and grade 6 signifies death. In this study, patients without disability were defined as having mRS scores of 0 or 1, while patients with disability had mRS scores of 2–5.

The Cerebral Performance Category (CPC) scale [38], was utilized to assess the neurological outcome and functional status of patients 28 days after CA. This scale comprises five categories: category 1 indicates good cerebral performance with normal function or minor disability allowing independent daily activities, category 2 represents moderate cerebral disability with some limitations but independent functioning in most daily activities, category 3 signifies severe cerebral disability requiring dependence on others for daily activities, category 4 reflects coma or vegetative state with unresponsiveness and unawareness of surroundings, and category 5 signifies brain death or death with no signs of brain function. In this study, good neurological status was defined as having CPC scores of 1 or 2, while poor neurological status was defined as having CPC scores of 3 or 4.

### Outcomes

The primary outcome examined was the 28-day survival rate following CA with favorable disability and neurological status. Additionally, several secondary outcomes were evaluated, including the 24-hour survival, ECMO survival (it indicates that these patients did not experience mortality while on ECMO therapy and does not encompass the final long-term outcome of the patients after ECMO support), CPR duration, ECMO length of stay (LOS), ICU LOS, and hospital LOS.

### Statistical analysis

Data were expressed as either means ± standard division (SD) or medians (inter-quartile range, IQR) for continuous variables, and frequencies with percentages (%) for categorical characteristics. The Shapiro-Wilk test was used to determine if the data followed a normal distribution.

Demographic and clinical characteristics of the patients were compared based on the NFT and LFT, where the division was made using the median values of NFT (≤ 5 min or > 5 min) and LFT (≤ 36 min or > 36 min). This approach of dividing the groups based on the median values allowed for a meaningful analysis and comparison of outcomes between patients with shorter and longer durations of NFT and LFT. For continuous variables, t-tests or Mann-Whitney tests were used, while for categorical variables, Chi-square or Fisher’s exact tests were employed. Additionally, unadjusted and adjusted binary logistic regression analyses were conducted to evaluate the prognostic clinical outcomes associated with NFT and LFT. The results were presented as odds ratios (OR) with 95% confidence intervals (95% CI).

To assess the predictive prognostic accuracy of NFT and LFT, receiver operating characteristic (ROC) curves and the corresponding area under the curves (AUC) were calculated. The AUC values were interpreted based on general guidelines: AUC between 0.9 and 1.0 indicated excellent discriminative power, 0.8–0.9 indicated good power, 0.7–0.8 indicated fair power, and 0.6–0.7 indicated poor power. Sensitivity (SN), specificity (SP), positive likelihood ratio (LR+), negative likelihood ratio (LR-), and Youden index were also considered to determine appropriate cut-off points. The AUCs between NFT and LFT were compared using the DeLong test.

Statistical analyses were performed using SPSS software (version 21) from SPSS Inc. (IL, Chicago, USA), GraphPad Prism 9© from GraphPad Software Inc. (La Jolla, CA), and MedCalc software. A significance level of 0.05 was used for all analyses.

## Results

### Demographic and clinical characteristics

This study included a total of 48 participants, with an average age of 41.88 ± 11.5 years. The majority of participants (75%) were male, and most (62.5%) came from the Asian/South Asian region, while 37.5% were from the Middle-East/Africa region. The participants had an average body mass index of 26.52 ± 5.23, indicating they were in the overweight range. The mean scores for SOFA and APACHE II were 12.21 ± 3.74 and 29.44 ± 7.62, respectively. Around 39.6% of participants had comorbidities, with ischemic heart disease being the most common (12.5%). OHCA occurred more frequently (77.1%) than IHCA (22.9%). Bystander-witnessed events and bystander CPR were reported in 93.8% and 97.9% of cases, respectively. Among the participants, 33.3% experienced an initial shockable rhythm during cardiac arrest.

In terms of resuscitation characteristics, the mean durations for NFT and LFT were 6.31 ± 3.37 min and 39.5 ± 19.63 min, respectively. The average time from collapse to ECPR was 45.81 ± 20.25 min. VA-ECMO was utilized in 91.7% of cases. However, in 8.3% of cases, VV-ECMO was chosen as these patients developed cardiac arrest in the context of severe acute respiratory distress syndrome (ARDS). The decision to use VV-ECMO in these patients was based on the presence of severe hypoxemia that ultimately led to cardiac arrest. Most ECPR procedures took place in the ED (68.8%), while the remainder occurred in the ICU (31.3%). Ischemic heart disease accounted for 43.8% of the cardiac arrest cases, followed by other causes (43.8%) and pulmonary embolism (12.5%). The median length of stay was 1.5 (1–4) days for ECMO, 3 (1-9.75) days for the ICU, and 4 (1–12) days for the hospital.

### Demographic and clinical outcomes according to NFT and LFT

Table 1 presents a comparison of demographic and clinical outcomes based on the duration of no-flow time (NFT): ≤5 min (*n* = 28, 58.3%) versus > 5 min (*n* = 20, 41.7%). When considering demographic factors, there were no significant differences observed in age, body mass index (BMI), severity of disease (based on SOFA and APACHE II scores), presence of comorbidities, or types of comorbidities between the two groups. However, there was a statistically significant difference in gender distribution, with the > 5 min group having a higher proportion of males compared to the NFT ≤ 5 min group (*P* = 0.042). Regarding clinical outcomes, the group with NFT > 5 min had a significantly longer duration of cardiopulmonary resuscitation (CPR) (59.25 ± 36.27 vs. 34.96 ± 23.63 min, *P* = 0.007), shorter median extracorporeal membrane oxygenation (ECMO) length of stay (LOS) (1 vs. 3 days, *P* = 0.019), shorter intensive care unit (ICU) LOS (1 vs. 7.5 days, *P* = 0.002), and shorter hospital LOS (1 vs. 8.5 days, *P* = 0.001).

**Table 1:** Demographic and clinical outcomes characteristics of the patients stratified by CPR start time (NFT)

Variables and outcomes		Total (*n* = 48)	CPR start time; (NFT)		*P*-value
			**≤ 5 min** (*n* = 28)	**> 5 min** (*n* = 20)	
Age, (years) #	(Mean ± SD)	41.88 ± 11.5	40.39 ± 12.66	43.20 ± 9.83	0.506
Gender, n (%) †	Male	36 (75)	18 (64.3)	18 (90)	0.042*
	Female	12 (25)	10 (35.7)	2 (10)	
Nationality, n (%) †	Middle-East/Africa	18 (37.5)	12 (42.9)	6 (30)	0.364
	Asian/South Asian	30 (62.5)	16 (57.1)	14 (70)	
Body Mass Index #	(Mean ± SD)	26.52 ± 5.23	26.82 ± 6.06	26.11 ± 3.91	0.650
Severity of disease,	SOFA score	12.21 ± 3.74	12.21 ± 3.45	12.20 ± 4.2	0.990
(Mean ± SD) #	APACHE II score	29.44 ± 7.62	29.54 ± 7.62	29.30 ± 7.82	0.917
Comorbidities †	yes (%)	19 (39.6)	12 (42.9)	7 (35)	0.583
Types of comorbidities †	Prior Ischemic heart disease	6 (12.5)	5 (17.9)	1 (5)	0.191
	Dyslipidemia	3 (6.3)	3 (10.7)	0	0.189
	Diabetes	10 (20.8)	4 (14.3)	6 (30)	0.168
	Hypertension	7 (14.6)	6 (21.4)	1 (5)	0.118
	Congestive heart failure	1 (2.1)	1 (3.6)	0	0.583
	Chronic respiratory disease	3 (6.3)	3 (10.7)	0	0.189
Cardiac arrest location †	In hospital (IHCA)	11 (22.9)	11 (39.3)	0	0.001*
	Out-of-hospital (OHCA)	37 (77.1)	17 (60.7)	20 (100)	
Bystander-witnessed †	yes (%)	45 (93.8)	27 (96.4)	18 (90)	0.364
Bystander CPR †	yes (%)	47 (97.9)	28 (100)	19 (95)	0.232
Initial shockable rhythm †	yes (%)	16 (33.3)	15 (53.6)	1 (5)	< 0.001*
Collapse-to-ECPR #	(Mean ± SD)	45.81 ± 20.23	40.25 ± 20.60	53.60 ± 17.34	0.023*
CPR-to-ECPR (LF time) #	(Mean ± SD)	39.5 ± 19.63	36.43 ± 20.33	43.80 ± 18.24	0.203
Duration of CPR	< 30 min (%)	16 (33.3)	13 (46.4)	3 (15)	0.023*
	> 30 min (%)	32 (66.7)	15 (53.6)	17 (85)	
CPR duration (min) #	(Mean ± SD)	45.08 ± 31.61	34.96 ± 23.63	59.25 ± 36.27	0.007*
Type of ECMO †	Venovenous (VV)	4 (8.3)	1 (3.6)	3 (15)	0.189
	Venoarterial (VA)	44 (91.7)	27 (96.4)	17 (85)	
ECPR location †	Emergency department (ED)	33 (68.8)	17 (60.7)	16 (80)	0.134
	Intensive Care Unit (ICU)	15 (31.3)	11 (39.9)	4 (20)	
Cause of cardiac arrest †	Ischemic heart disease	21 (43.8)	13 (46.4)	8 (40)	0.863
	Pulmonary embolism	6 (12.5)	3 (10.7)	3 (15)	
	Others	21 (43.8)	12 (42.9)	9 (45)	
ECMO length of stay ‡	Median (IQR)	1.5 (1–4)	3 (1–5)	1 (1–2)	0.019*
ICU length of stay ‡	Median (IQR)	3 (1-9.75)	7.5 (1.25–20.75)	1 (1–3)	0.002*
Hospital length of stay‡	Median (IQR)	4 (1–12)	8.5 (2-35.75)	1 (1–4)	0.001*
28-day disability status †	No disability survivors	5 (10.4)	5 (17.9)	0	0.001*
	Disability survivors	9 (18.8)	9 (32.1)	0	
	Non-survivors	34 (70.8)	14 (50)	20 (100)	
28-day neurological status †	Good recovery survivors	9 (18.8)	9 (32.1)	0	0.001*
	Poor recovery survivors	5 (10.4)	5 (17.9)	0	
	Non-survivors	34 (70.8)	14 (50)	20 (100)	
ECMO Survival †	yes (%)	17 (35.4)	16 (57.1)	1 (5)	< 0.001*
24-h Survival †	yes (%)	31 (64.6)	22 (78.9)	9 (45)	0.017*
28-day Survival †	yes (%)	14 (29.2)	14 (50)	0	< 0.001*

* *P* < 0.05 considered as significantly, # t-test, † Chi-square test or Fisher exact test, ‡ Mann-Whitney test, Sequential Organ Failure Assessment (SOFA) Score, Acute Physiology and Chronic Health Evaluation (APACHE II), Cardiopulmonary resuscitation (CPR), Extracorporeal cardiopulmonary resuscitation (ECPR), Extracorporeal membrane oxygenation (ECMO), No flow time (NFT).

Table 2 presents a comparison of demographic and clinical outcomes based on the duration of low-flow time (LFT): ≤36 min and > 36 min. Regarding demographic characteristics, no significant differences were observed in age, gender, nationality, or the presence of comorbidities between the two groups. However, the group with LFT > 36 min had a significantly higher BMI compared to the group with LFT ≤ 36 min (28.19 ± 5.86 vs. 24.85 ± 3.98, *P* = 0.026). In terms of clinical outcomes, the duration of CPR was significantly longer in the LFT > 36 min group (57.75 ± 24.13 vs. 32.42 ± 33.54 min, *P* = 0.004), as well as the longer time from collapse to ECMO (61.08 ± 15.60 vs. 30.54 ± 10.33 min, *P* < 0.001), compared to the LFT ≤ 36 min group. There were no statistically significant differences observed between the two groups in terms of other clinical outcomes such as severity of disease, type of cardiac arrest, ECMO LOS, ICU LOS, or hospital LOS.

**Table 2:** Demographic and clinical outcomes characteristics of the patients stratified by CPR to ECPR time (LFT)

Variables and outcomes		Total (*n* = 48)	CPR to ECRP; (LFT)		*P*-value
			**≤ 36 min** (*n* = 24)	**> 36 min** (*n* = 24)	
Age, (years) #	(Mean ± SD)	41.88 ± 11.5	43.08 ± 12.55	40.67 ± 10.48	0.473
Gender, n (%) †	Male	36 (75)	16 (66.7)	20 (83.3)	0.159
	Female	12 (25)	8 (33.3)	4 (16.7)	
Nationality, n (%) †	Middle-East/Africa	18 (37.5)	9 (37.5)	9 (37.5)	0.999
	Asian/South Asian	30 (62.5)	15 (62.5)	15 (62.5)	
Body Mass Index #	(Mean ± SD)	26.52 ± 5.23	24.85 ± 3.98	28.19 ± 5.86	0.026*
Severity of disease,	SOFA score	12.21 ± 3.74	12.04 ± 3.22	12.38 ± 4.26	0.761
(Mean ± SD) #	APACHE II score	29.44 ± 7.62	29.17 ± 8.24	29.71 ± 7.12	0.809
Comorbidities †	yes (%)	19 (39.6)	9 (37.5)	10 (41.7)	0.768
Types of comorbidities †	Prior Ischemic heart disease	6 (12.5)	2 (8.3)	4 (16.7)	0.333
	Dyslipidemia	3 (6.3)	2 (8.3)	1 (4.2)	0.989
	Diabetes	10 (20.8)	4 (16.7)	6 (25)	0.362
	Hypertension	7 (14.6)	5 (20.8)	2 (8.3)	0.208
	Congestive heart failure	1 (2.1)	0	1 (4.2)	0.877
	Chronic respiratory disease	3 (6.3)	2 (8.3)	1 (4.2)	0.896
Cardiac arrest location †	In hospital (IHCA)	11 (22.9)	7 (29.2)	4 (16.7)	0.247
	Out-of-hospital (OHCA)	37 (77.1)	17 (70.8)	20 (83.3)	
Bystander-witnessed †	yes (%)	45 (93.8)	22 (91.7)	23 (95.8)	0.551
Bystander CPR †	yes (%)	47 (97.9)	23 (95.8)	24 (100)	0.312
Initial shockable rhythm †	yes (%)	16 (33.3)	11 (45.8)	5 (20.8)	0.066
Collapse-to-CPR (NF time) #	(Mean ± SD)	6.31 ± 3.37	5.96 ± 3.74	6.67 ± 2.98	0.473
Duration of CPR †	< 30 min (%)	16 (33.3)	13 (54.2)	3 (12.5)	0.002*
	> 30 min (%)	32 (66.7)	11 (45.8)	21 (87.5)	
CPR duration (min) #	(Mean ± SD)	45.08 ± 31.61	32.42 ± 33.54	57.75 ± 24.13	0.004*
Collapse-to-ECPR #	(Mean ± SD)	45.81 ± 20.25	30.54 ± 10.33	61.08 ± 15.60	< 0.001*
Type of ECMO †	Venovenous (VV)	4 (8.3)	3 (12.5)	1 (4.2)	0.609
	Venoarterial (VA)	44 (91.7)	21 (87.5)	23 (95.8)	
ECPR location †	Emergency department (ED)	33 (68.8)	14 (58.3)	19 (79.2)	0.106
	Intensive Care Unit (ICU)	15 (31.3)	10 (41.7)	5 (20.8)	
Cause of cardiac arrest †	Ischemic heart disease	21 (43.8)	9 (37.5)	12 (50)	0.319
	Pulmonary embolism	6 (12.5)	2 (8.3)	4 (16.7)	
	Others	21 (43.8)	13 (54.2)	8 (33.3)	
ECMO length of stay ‡	Median (IQR)	1.5 (1–4)	3 (1-5.75)	1 (1-2.75)	0.079
ICU length of stay ‡	Median (IQR)	3 (1-9.75)	6 (1-14.25)	1.5 (1-6.75)	0.199
Hospital length of stay ‡	Median (IQR)	4 (1–12)	6 (1-25.75)	1.5 (1-8.75)	0.157
28-day disability status †	No disability survivors	5 (10.4)	3 (12.5)	2 (8.3)	0.807
	Disability survivors	9 (18.8)	5 (20.8)	4 (16.7)	
	Non-survivors	34 (70.8)	16 (66.7)	18 (75)	
28-day neurological status†	Good recovery survivors	9 (18.8)	5 (20.8)	4 (16.7)	0.807
	Poor recovery survivors	5 (10.4)	3 (12.5)	2 (8.3)	
	Non-survivors	34 (70.8)	16 (66.7)	18 (75)	
ECMO Survival †	yes (%)	17 (35.4)	11 (45.8)	6 (25)	0.131
24-h Survival †	yes (%)	31 (64.6)	16 (66.7)	15 (62.5)	0.763
28-day Survival †	yes (%)	14 (29.2)	8 (33.3)	6 (25)	0.525

* *P* < 0.05 considered as significantly, # t-test, † Chi-square test or Fisher exact test, ‡ Mann-Whitney test, Sequential Organ Failure Assessment (SOFA) Score, Acute Physiology and Chronic Health Evaluation (APACHE II), Cardiopulmonary resuscitation (CPR), Extracorporeal cardiopulmonary resuscitation (ECPR), Extracorporeal membrane oxygenation (ECMO), Low flow time (LFT).

### Survival and neurological outcomes according to NFT and LFT

The survival rate within 24 h after CA was found to be 64.4%. However, this rate significantly decreased to 35.4% at the time of extracorporeal membrane oxygenation (ECMO), and further decreased to 29.2% at 28 days after CA. Out of the 14 patients who survived beyond 28 days after the cardiac arrest, 9 (64.3%) of them had a favorable neurological status (CPC 1–2), indicating good recovery. Additionally, 5 (35.7%) patients had no disability, as indicated by a mRS of 0–1.

The study revealed a significant difference in survival rates based on the duration of NFT (Table 1). The group with NFT > 5 min had lower survival rates compared to the group with NFT ≤ 5 min. Specifically, on ECMO, the survival rates were 5% and 57.1% respectively (*P* < 0.001). At 24 h, the survival rates were 45% and 78.9% respectively (*P* = 0.017). At 28 days, the survival rates were 0% and 50% respectively (*P* < 0.001). These findings indicate the importance of NFT duration in determining survival outcomes. Additionally, the group with NFT > 5 min had no survivors without disability and no survivors with good recovery, while the group with NFT ≤ 5 min had 5 survivors without disability and 9 survivors with good recovery (*P* = 0.001). Both groups had 5 survivors with poor recovery.

Based on the duration of LFT (Table 2), there were no statistically significant differences between the ≤ 36 min and > 36 min groups in terms of 28-day disability status (10.4% vs. 12.5%, *p* = 0.807) and 28-day neurological status (18.8% vs. 20.8%, *p* = 0.807). The ECMO survival rates were 35.4% in the total cohort, 45.8% in the ≤ 36 min group, and 25% in the > 36 min group, but the difference was not significant (*p* = 0.131). The 24-hour and 28-day survival rates showed no significant variation between the two groups (*p* = 0.763 and *p* = 0.525, respectively).

### Prognostic impact of NFT and LFT on clinical outcomes

Figure 1 presents the results of binary logistic regression analyses, both unadjusted and adjusted, for age, gender, BMI, and cardiac arrest location (in-hospital or out of hospital). The objective of these analyses was to determine the prognostic clinical outcomes associated with NFT and LFT.

**Fig. 2:**
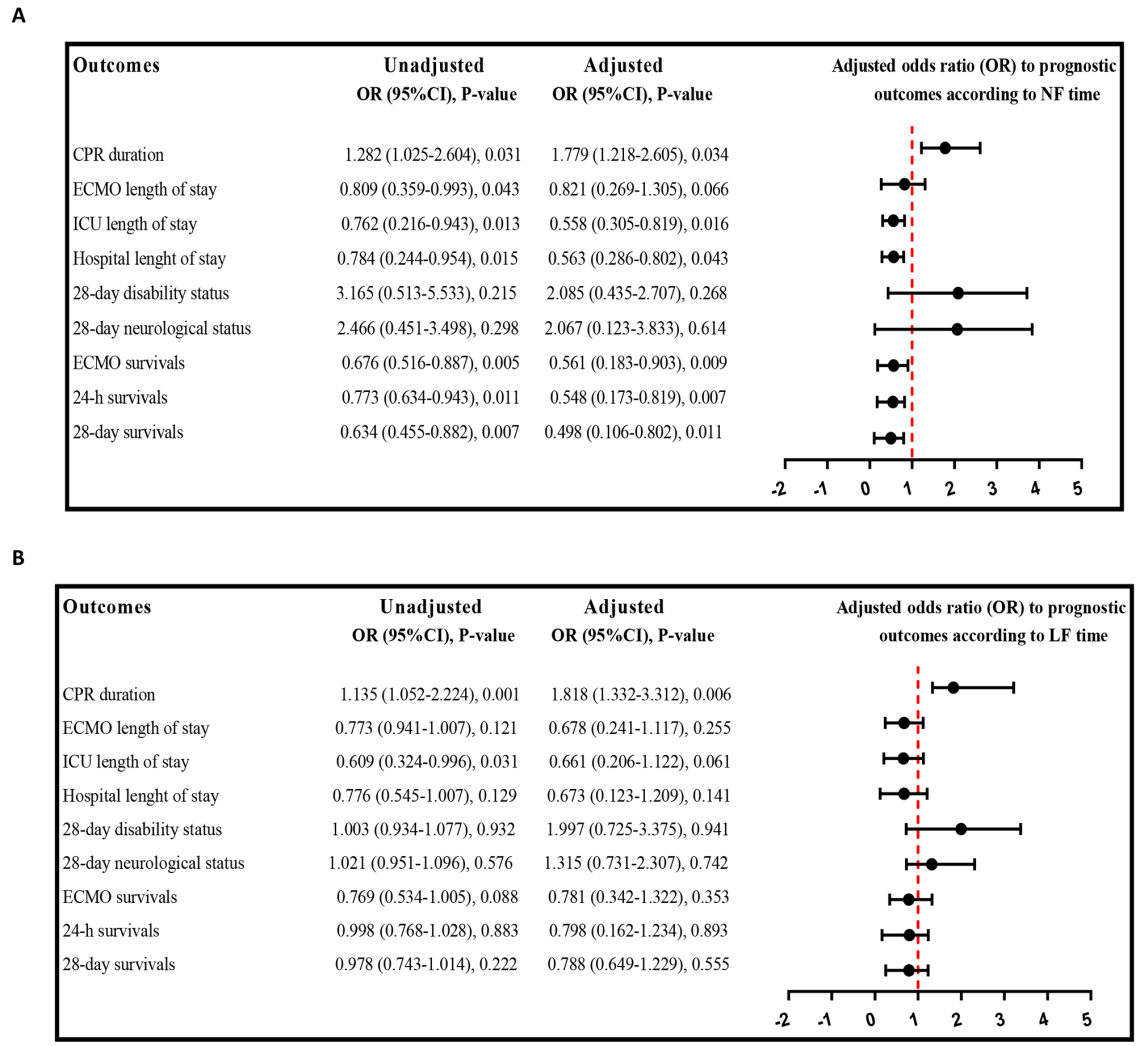
Unadjusted and adjusted (adjusting by age, gender BMI and cardiac arrest location include in-hospital or out of hospital) binary logistic regression analysis to prognostic clinical outcomes according to **(A)** CPR start time (NFT: No Flow time), and **(B)** CPR to ECPR time (LFT: Low Flow time). Forest plot showed adjusted odd ratios (ORs) to prognostic outcomes according NF time and LF time. P<0.05 considered as significantly

In the adjusted regression analysis, longer NFT was found to significantly decrease the risk of ICU LOS (OR: 0.558, 95%CI: 0.305–0.819, *P* = 0.016), hospital LOS (OR: 0.563, 95%CI: 0.286-0.80, *P* = 0.043), ECMO survival (OR: 0.561, 95%CI: 0.183–0.903, *P* = 0.009), 24-h survival (OR: 0.548, 95%CI: 0.173–0.819, *P* = 0.007), and 28-day survival (OR: 0.498, 95%CI: 0.106–0.802, *P* = 0.011). However, longer NFT was found to significantly increase the risk of longer CPR duration (> 30 min) (OR: 1.779, 95%CI: 1.218–2.605, *P* = 0.034). On the other hand, NFT was not significantly associated with changes in the odds ratio for 28-day disability status (OR: 2.085, 95%CI: 0.435–2.707, *P* = 0.268) and 28-day neurological status (OR: 2.067, 95%CI: 0.123–3.833, *P* = 0.614) (Fig. 2A).

In contrast to NFT, LFT showed no significant association with changes in the odds ratio for ICU LOS, hospital LOS, ECMO survival, 24-hour survival, and 28-day survival based on adjusted regression analysis. However, longer LFT was found to significantly increase the risk of prolonged CPR duration (> 30 min) (OR: 1.818, 95%CI: 1.332–3.312, *P* = 0.006). Similar to NFT, LFT was also not significantly associated with changes in the odds ratio for 28-day disability status (OR: 1.997, 95%CI: 0.725–3.375, *P* = 0.941) and 28-day neurological status (OR: 1.315, 95%CI: 0.731–2.307, *P* = 0.742) (Fig. 2B).

### Predicting clinical outcomes by NFT and LFT

Predicting clinical outcomes using NFT and LFT was examined and the results are presented in Table 3. In terms of NFT, the AUC values ranged from 0.669 to 0.776, indicating moderate to fair predictive accuracy for various factors. Specifically, NFT showed significant predictive value for ECMO LOS (AUC: 0.669, 95% CI: 0.519–0.798, *P* = 0.034), hospital LOS (AUC: 0.683, 95% CI: 0.533–0.810, *P* = 0.020), 24-hour survival (AUC: 0.714, 95% CI: 00.566–0.835, *P* = 0.011), ICU LOS (AUC: 0.717, 95% CI: 0.569–0.837, *P* = 0.003), CPR duration (AUC: 0.741, 95% CI: 0.595–0.857, *P* = 0.006), 28-day survival (AUC: 0.768, 95% CI: 0.624–0.877, *P* = 0.0001), and ECMO survival (AUC: 0.776, 95% CI: 0.633–0.884, *P* = 0.0001).

**Table 3:** ROC curve results of CPR start time (NFT) and CPR to ECPR time (LFT) to predict outcomes

	Outcomes	AUC (95% CI)	*P*-value	SN (95% CI)	SP (95% CI)	LR+ (95% CI)	LR- (95% CI)	Youden Index	Cut-point
CPR start time (NFT)	**CPR duration** (> 30 min vs. <30 min)	0.741 (0.595–0.857)	0.006*	50.00 (24.7–75.3)	93.75 (79.2–99.2)	8.00 (1.92–33.38)	0.53 (0.32–0.88)	0.437	≤ 3
	**ECMO length of stay** (≥ 2 days vs. <2 days)	0.669 (0.519–0.798)	0.034*	56.67 (37.4–74.5)	77.78 (52.4–93.6)	2.55 (1.02–6.39)	0.56 (0.35–0.90)	0.344	> 5
	**ICU length of stay** (≥ 3 days vs. <3 days)	0.717 (0.569–0.837)	0.003*	60.71 (40.6–78.5)	80.00 (56.3–94.3)	3.04 (1.20–7.66)	0.49 (0.29–0.82)	0.407	> 5
	**Hospital length of stay** (≥ 4 days vs. <4 days)	0.683 (0.533–0.810)	0.020*	62.50 (40.6–81.2)	75.00 (53.3–90.2)	2.50 (1.17–5.34)	0.50 (0.28–0.88)	0.375	> 5
	**28-day disability status** (with disability vs. without disability)	0.689 (0.394–0.901)	0.151	100 (47.8–100)	33.33 (7.5–70.1)	1.50 (0.95–2.38)	0	0.333	≤ 4
	**28-day neurological status** (poor status vs. good status)	0.656 (0.363–0.881)	0.335	88.89 (51.8–99.7)	40.00 (5.3–85.3)	1.48 (0.70–3.14)	0.28 (0.033–2.35)	0.288	≤ 4
	**ECMO survival** (yes vs. no)	0.776 (0.633–0.884)	0.0001*	94.12 (71.3–99.9)	64.52 (45.4–80.8)	2.65 (1.63–4.33)	0.091 (0.013–0.62)	0.586	≤ 5
	**24-h survival** (yes vs. no)	0.714 (0.566–0.835)	0.011*	77.42 (58.9–90.4)	70.59 (44.0-89.7)	2.63 (1.23–5.63)	0.32 (0.16–0.66)	0.480	≤ 7
	**28-day survival** (yes vs. no)	0.768 (0.624–0.877)	0.0001*	100 (76.8–100)	61.76 (43.6–77.8)	2.62 (1.71–4.01)	0	0.617	≤ 5
CPR to ECPR time (LFT)	**CPR duration** (> 30 min vs. <30 min)	0.878 (0.751–0.955)	0.0001*	75.00 (47.6–92.7)	93.75 (79.2–99.2)	12.00 (3.04–47.29)	0.27 (0.11–0.63)	0.687	≤ 27
	**ECMO length of stay** (≥ 2 days vs. <2 days)	0.635 (0.484–0.769)	0.096	60.00 (40.6–77.3)	66.67 (41.0-86.7)	1.80 (0.88–3.68)	0.60 (0.35–1.04)	0.266	> 36
	**ICU length of stay** (≥ 3 days vs. <3 days)	0.696 (0.547–0.821)	0.010*	64.29 (44.1–81.4)	70.00 (45.7–88.1)	2.14 (1.04–4.42)	0.51 (0.29–0.91)	0.342	> 36
	**Hospital length of stay** (≥ 4 days vs. <4 days)	0.640 (0.488–0.773)	0.085	62.50 (40.6–81.2)	62.50 (40.6–81.2)	1.67 (0.91–3.04)	0.60 (0.33–1.10)	0.250	> 36
	**28-day disability status** (with disability vs. without disability)	0.500 (0.230–0.770)	0.999	20.00 (0.5–71.6)	55.56 (21.2–86.3)	0.45 (0.067–3.01)	1.44 (0.69–2.99)	0.244	< 39
	**28-day neurological status** (poor status vs. good status)	0.578 (0.294–0.828)	0.647	33.33 (7.5–70.1)	100 (47.8–100)	0	0.67 (0.42–1.06)	0.333	≤ 17
	**ECMO survival** (yes vs. no)	0.646 (0.495–0.779)	0.075	47.06 (23.0-72.2)	77.42 (58.9–90.4)	2.08 (0.91–4.75)	0.68 (0.42–1.11)	0.244	≤ 28
	**24-h survival** (yes vs. no)	0.538 (0.388–0.683)	0.661	29.03 (14.2–48.0)	88.24 (63.6–98.5)	2.47 (0.60-10.14)	0.80 (0.61–1.07)	0.172	≤ 22
	**28-day survival** (yes vs. no)	0.599 (0.447–0.738)	0.273	42.86 (17.7–71.1)	76.47 (58.8–89.3)	1.82 (0.77–4.29)	0.75 (0.46–1.22)	0.193	≤ 27

Abbreviations; NFT: No flow time, LFT: Low flow time, CI: Confidence interval, SN: Sensitivity; SP: Specificity; LR+: Positive likelihood ratio; LR-: Negative likelihood ratio; *P-value < 0.05 considered significant

On the other hand, LFT demonstrated good predictive performance specifically in relation to CPR duration, with an area under the ROC curve of 0.878 (95% CI: 0.751–0.955, *P* = 0.0001). The optimal cut-off value for LFT was identified as ≤ 27 min, with a sensitivity of 75% (95%CI: 47.6-92.7%), specificity of 93.7% (95%CI: 79.2–99.2), a positive likelihood ratio (LR+) of 12 (95%CI: 3.04–47.29), a negative likelihood ratio (LR-) of 0.27 (95%CI: 0.11–0.63), and a Youden index of 0.687%.

### Predictive accuracy analysis: NFT vs. LFT

**Fig. 3:**
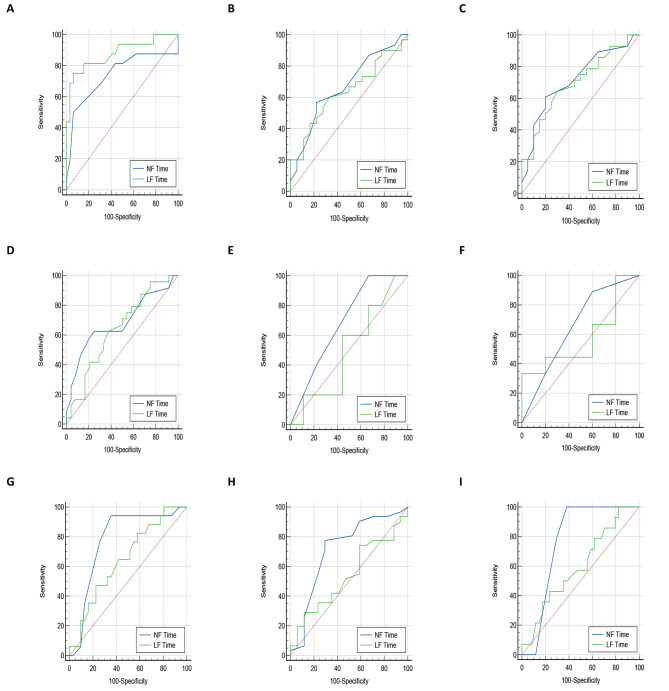
Comparison of ROC curves between NFT and LFT to predict **(A)** CPR duration, **(B)** ECMO LOS, **(C)** ICU LOS, **(D)** hospital LOS, **(E)** 28-day disability status, **(F)** 28-day neurological status, **(G)** ECMO survival, **(H)** 24-h survival, and **(I)** 28-day surviva

The comparative analysis between NFT and LFT in predicting various outcomes is presented in Table 4. The results indicate that, overall, NFT exhibits higher predictive accuracy than LFT for length of stay and survival outcomes. However, in the specific case of predicting CPR duration, LFT performs better than NFT. Importantly, the statistical analysis using the DeLong test did not reveal any significant differences between NFT and LFT in terms of predictive accuracy. For a visual comparison, refer to Fig. 3 which illustrates the ROC curves of NFT and LFT in predicting clinical outcomes.

**Table 4:** Comparison of AUC between CPR start time (NFT) and CPR to ECPR time (LFT) to predict outcomes

Outcomes	CPR start time (NFT)			CPR to ECPR time (LFT)			p-value*
	AUC	95% CI	p-value	AUC	95% CI	p-value	
CPR duration	0.741	0.595 to 0.857	0.006	0.878	0.751 to 0.955	0.0001	0.178
ECMO length of stay	0.669	0.519 to 0.798	0.034	0.635	0.484 to 0.769	0.096	0.743
ICU length of stay	0.717	0.569 to 0.837	0.003	0.696	0.547 to 0.821	0.010	0.835
Hospital length of stay	0.683	0.533 to 0.810	0.020	0.640	0.488 to 0.773	0.085	0.672
28-day disability status	0.689	0.394 to 0.901	0.151	0.500	0.230 to 0.770	0.999	0.488
28-day neurological status	0.656	0.363 to 0.881	0.335	0.578	0.294 to 0.828	0.647	0.654
ECMO survival	0.776	0.633 to 0.884	0.0001	0.646	0.495 to 0.779	0.075	0.188
24-h survival	0.714	0.566 to 0.835	0.011	0.538	0.388 to 0.683	0.661	0.094
28-day survival	0.768	0.624 to 0.877	0.0001	0.599	0.447 to 0.738	0.273	0.085

* P-value based on DeLong test to compare AUCs between NFT and LFT for each outcome

## Discussion

Despite advancements in resuscitation techniques and knowledge, the treatment of OHCA and IHCA remains challenging, leading to consistently low survival rates and poor neurological status upon discharge [39]. Minimizing collapse-to-CPR time (NFT) and CPR-to-ECPR time (LFT) is crucial for improving outcomes in OHCA and IHCA patients [36, 40]. This study aimed to assess the impact of NFT and LFT on prognosis in patients undergoing ECMO. The results clearly showed a significant relationship between these time intervals and outcomes in OHCA and IHCA patients.

The findings indicated that NFT was a more effective predictor than LFT in assessing clinical outcomes for OHCA and IHCA patients who underwent ECMO. Longer NFT was associated with unfavorable outcomes, including longer CPR duration and decreased survival rates for ECMO, 24 h, and 28 days. The effectiveness of NFT as a predictor of clinical outcomes in OHCA and IHCA patients undergoing ECMO can be attributed to the early initiation of CPR, timely restoration of circulation, and the influence of resuscitation quality. Minimizing NFT intervals through prompt recognition and initiation of CPR increases the likelihood of successful resuscitation and improved survival rates. These findings align with previous studies that reported an inverse correlation between NFT and survival rate. A study by Hasselqvist-Ax et al. [41], analyzed data from the Swedish Cardiac Arrest Registry involving 30,381 OHCA patients and demonstrated that a shorter NFT was associated with a more than twofold higher 30-day survival rate compared to a longer NFT. Reynolds et al. [42], found that the probability of survival to the hospital for ECPR patients decreased rapidly with each minute of delayed onset CPR. Additionally, a single-center retrospective study on 85 patients who received ECPR indicated that an NFT ≤ 5 min was a superior predictor for favorable outcomes in OHCA patients compared to the combined NFT and LFT [21].

Optimal NFT cut-off values of ≤ 7 min and ≤ 5 min were identified for predicting 24-hour survival and 28-day survival, respectively. Notably, all participants who survived beyond 28 days had a collapse-to-CPR time of less than 5 min. The identified optimal NFT cut-off values suggest that prompt initiation of CPR within these time frames maximizes the chances of successful resuscitation and subsequent survival. Furthermore, the finding that all participants who survived beyond 28 days had a collapse-to-CPR time of less than 5 min emphasizes the critical importance of early CPR in achieving long-term favorable outcomes.

Regarding LFT, a longer duration was associated with a higher probability of prolonged CPR but was not an independent predictor of survival in this study. However, prolonged CPR duration is known to decrease survival rates due to complications and diminishing CPR effectiveness. Prolonged CPR duration can have negative effects on survival rates after cardiac arrest (CA). Firstly, it increases the risk of complications, such as brain damage and permanent disability, due to the prolonged period of inadequate blood flow to the brain. Additionally, as time goes on, the effectiveness of CPR diminishes, leading to decreased chances of successful resuscitation and subsequent survival [43]. Goto et al. [16], in a prospective observational study revealed an independent and inverse association between the duration of CPR and the 1-month survival and favorable neurological outcomes in Japanese OHCA patients. Another study by Lee et al. [44], examined 605 patients who experienced IHCA and found that the median CPR duration was 11.0 min for the survival group and 26.5 min for the non-survival group. Wang et al. [45], conducted a study that indicated a trend towards lower rates of favorable outcomes in patients with longer LFT. Higashi et al. [40], performed a study on 117 IHCA subjects who received ECPR and found an inverse correlation between LFT and the 90-day survival rate as well as favorable neurological outcomes. Therefore, it is evident that longer LFT durations are associated with poorer survival outcomes [46, 47]. Additionally, the most recent meta-analysis demonstrated that a shorter LFT in ECPR was linked to improved survival [48]. Considering the upper limit of CPR duration (between 21 and 34 min) needed to achieve favorable neurologic outcomes [49], we determined that the optimal LFT cut-off value for predicting durations below 30 min was ≤ 27 min.

The study found no statistically significant connection between NFT or LFT and the improvement of disability and neurologically favorable survival after 28 days of cardiac arrest. However, it is worth noting that the NFT was shorter in patients who survived for 28 days with good neurological status compared to those with poor neurological status. Although not statistically significant, this finding aligns with previous studies that reported an inverse relationship between NFT/LFT and favorable neurological outcomes [16, 21, 23]. Further research is needed to understand the exact nature of this correlation, considering factors such as sample size, patient characteristics, and treatment protocols. Future studies may explore interventions to decrease NFT and LFT while enhancing neurological outcomes following cardiac arrest.

### Strengths and limitations

The findings of our study offer valuable insights into the predictive abilities of NFT and LFT regarding clinical outcomes in patients with OHCA and IHCA. These insights can guide healthcare professionals in enhancing resuscitation protocols, which have potential to improve survival rates and long-term prognosis for patients undergoing ECMO. However, there are some potential limitations in the present observational study. First, the study has a single-center retrospective design, which may limit the generalizability of the findings. Second, the small sample size can influence the accuracy and reliability of the results, leading to conflicting conclusions. Third, the study relies on reports of estimated collapse times. However, the accuracy and reliability of these estimates may vary, potentially introducing measurement errors or inconsistencies into the data. Fourth, there is a lack of previous information available on the neurological status of patients prior to cardiac arrest. This absence of data could affect the interpretation of the results. Nevertheless, the study attempted to address these limitations by utilizing strict inclusion and exclusion criteria to select homogeneous participants and reduce the effect of confounding factors using adjusted binary regression analysis, thereby enhancing the internal validity and generalizability of the study findings.

## Conclusion

The results indicate that a longer NFT is strongly associated with worse clinical outcomes, as measured by various parameters. Patients with an extended NFT had significantly longer CPR duration, suggesting a more challenging resuscitation process. Additionally, there was an inverse correlation between NFT and ECMO survival, 24-hour survival, and 28-day survival, highlighting its potential as an indicator of adverse outcomes. In other words, the analysis revealed that a longer LFT was only linked to an increased risk of prolonged CPR duration. This implies that while LFT may offer some predictive value for CPR duration, it lacks the comprehensive predictive power demonstrated by NFT concerning overall clinical outcomes. Therefore, our study presents compelling evidence supporting NFT as a superior predictor compared to LFT for clinical outcomes in OHCA or IHCA patients undergoing ECMO. Understanding these distinct predictive abilities allows medical professionals to better identify high-risk patients and tailor their interventions accordingly. It is important to note that further research is required to explore the underlying mechanisms behind the associations observed in our study. Future investigations could delve into the specific physiological and pathophysiological factors influencing NFT and LFT, providing insights into the reasons for their discrepant predictive performances.

## Data Availability

The data that support the findings of this study are available from the corresponding author upon reasonable request.

## List of Abbreviations

CPR: Cardiopulmonary resuscitation
ECPR: Extracorporeal cardiopulmonary resuscitation
ROSC: Return of spontaneous circulation
OHCA: Out of-hospital cardiac arrest
IHCA: In hospital cardiac arrest
NFT: No flow time
LFT: Low flow time
ECMO: Extracorporeal membrane oxygenation
CA: Cardiac arrest
CPC: Cerebral Performance Category
mRS: modified Rankin Scale
VV-ECMO: Veno-venous ECMO
VA-ECMO: Veno-arterial ECMO
OR: Odds ratio
CI: Confidence intervals
ROC: Receiver operating characteristic
